# Advances in the Treatment of Striae Distensae

**DOI:** 10.1111/jocd.70683

**Published:** 2026-01-20

**Authors:** Yi Wu, Huaigu Wang

**Affiliations:** ^1^ Department of Plastic and Burn Surgery The First Affiliated Hospital of Bengbu Medical University Bengbu Anhui China

**Keywords:** collagen remodeling, non‐ablative fractional laser, skin regeneration, Striae distensae

## Abstract

**Background:**

Striae distensae (SD) is a common cutaneous condition with an estimated incidence of 56%, associated with rapid skin expansion, hormonal changes, and genetic factors. While traditional treatments (e.g., topical tretinoin, various lasers) exhibit limitations, non‐ablative fractional laser (NAFL), injectable fillers, alpha hydroxy acids (AHAs), and combination therapies have emerged as promising modalities, though certain aspects require clarification.

**Aims:**

This review aims to synthesize current evidence on the pathogenesis and management of SD, with a particular focus on evaluating the recent progress of 1565 nm NAFL to provide guidance for clinical practice and future research.

**Methods:**

A systematic literature search was conducted across PubMed, Web of Science, and Scopus databases following the Preferred Reporting Items for Systematic Reviews and Meta‐Analyses (PRISMA) guidelines. The review prioritized randomized controlled trials, meta‐analyses, and substantial clinical studies published between 2010 and 2025.

**Results:**

Striae distensae (SD) arises from mechanical tension, hormonal imbalance, and inflammation‐induced dermal matrix disruption. Efficacious treatments include alpha hydroxy acids, injectable fillers, 1540 nm Er:Glass laser, 532 nm Nd:YAG laser, picosecond lasers, intense pulsed light, and combination therapies (e.g., fractional microneedling/CO_2_ laser + PRP). 1565 nm NAFL exhibits favorable efficacy, safety, and tolerance, with enhanced outcomes in combinations.

**Conclusions:**

The management of SD is evolving towards individualized combination strategies. Fractional microneedling plus PRP and fractional CO_2_ laser resurfacing with PRP achieve good results in SD rubra and alba, while 1565 nm NAFL represents a significant advance in minimally invasive treatment. Future research should prioritize large‐scale studies, standardized assessment methods, and exploration of novel combinations.

## 1 Introduction

Striae distensae (SD), also known as stretch marks or striae atrophicae, is a common cutaneous condition characterized by linear atrophy. Its pathogenesis is closely related to rapid skin expansion, hormonal fluctuations, and genetic susceptibility [1, 2]. Epidemiological data show that the overall incidence of SD is 56% (95% CI 52%–59%) [3]. It not only causes abnormal skin texture and color but also triggers psychological problems such as anxiety and depression due to its significant impact on appearance [4, 5]. Pathologically, SD is manifested as the rupture of collagen fibers, a decrease in elastic fibers in the dermis, and epidermal atrophy. Its development can be divided into early inflammatory red striae (Striae Rubrae) and late fibrotic white striae (Striae Albae) [6, 7]. The pathological features at different stages provide an important basis for the selection of treatment strategies.

Traditional treatment methods include topical tretinoin, microneedle radiofrequency, platelet‐rich plasma (PRP), and laser technology. However, the curative effects vary significantly and have limitations. For example, although microneedle radiofrequency can promote collagen remodeling, it causes obvious pain [8, 9]. The CO_2_ fractional laser is effective for white striae, but ablative injury is likely to lead to abnormal pigmentation and a prolonged recovery period [10].

In recent years, non‐ablative fractional laser (NAFL) has attracted much attention due to its unique thermal stimulation effect and minimally invasive advantages. This technology forms microthermal zones (MTZs) in the middle dermis through targeted action, activates fibroblast proliferation, and induces collagen neogenesis; at the same time, it preserves the integrity of the epidermis to reduce the risk of adverse reactions [8, 11, 12]. Clinical studies have shown that 1565 nm NAFL alone can significantly improve the atrophy and appearance of stretch marks, and combination therapies (such as combined with β‐glucan) can further enhance the efficacy [11].

However, there is still no consensus on the mechanism of action, long‐term efficacy of NAFL, and its synergistic effects with other therapies. This article reviews the pathogenesis of SD and existing treatment methods, aiming to provide a basis for clinical practice and scientific research directions.

## 2 Methods

This review article was conducted following Preferred Reporting Items for Systematic Reviews and Meta‐Analyses (PRISMA) guidelines—a standard methodological framework for high‐quality review articles—to ensure transparency and rigor in literature selection and data synthesis. The PubMed database was primarily used with the search term “(Striae Distensae[MeSH Terms] OR SD OR **‘**Stretch Marks**’** OR **‘**Striae Rubrae**’** OR **‘**Striae Albae**’** OR **‘**Striae Gravidarum**’**) AND (**‘**Treatment**’** OR **‘**Therapeutic Progress**’** OR **‘**Combination Therapy**’**),”. Additional databases such as Web of Science and Scopus were also scanned to ensure comprehensiveness and minimize publication bias, consistent with PRISMA recommendations.

Inclusion criteria focused on peer‐reviewed studies published in English between 2010 and 2025, including randomized controlled trials, meta‐analyses, and review articles that discussed therapeutic advances for SD. Exclusion criteria comprised non‐human studies, case reports with sample sizes smaller than 10, articles not focused on treatment efficacy, and duplicates.

The study selection process followed a two‐step screening procedure to strictly assess relevance and eligibility:

- Title/abstract screening: Two independent reviewers first evaluated the titles and abstracts of all retrieved publications against the predefined inclusion and exclusion criteria. This step aimed to eliminate clearly irrelevant studies (e.g., those focusing solely on SD pathogenesis without treatment data, non‐English articles) to reduce the volume of full texts requiring further review. Discrepancies at this stage were resolved through discussion between the two reviewers, with a third reviewer consulted if consensus could not be reached.
- Full‐text review: Publications deemed potentially eligible after title/abstract screening underwent detailed full‐text assessment to confirm adherence to all inclusion criteria. Studies were excluded at this stage if they lacked clear efficacy metrics (e.g., no quantitative reduction in striae width) or failed to report key treatment parameters (e.g., laser energy density, drug concentration).

Data extraction was performed using a standardized form, capturing detailed categories as follows: to ensure transparency and reproducibility:

- Study characteristics: First author, publication year, study design (e.g., RCT, cohort study, meta‐analysis), countries/regions of participant recruitment, total sample size, mean participant age (± standard deviation), gender distribution (percentage of male/female participants), and type of SD (Striae Rubrae/Striae Albae, diagnosed via clinical criteria such as color and texture).
- Treatment of striae: Modality type (e.g., topical drug, microneedling, laser), use of monotherapy or combination therapy (with specific combination partners listed), delivery mode (e.g., topical application frequency, intradermal injection intervals), key parameters (e.g., drug concentration, laser wavelength, microneedle depth), total treatment duration, and follow‐up period (with specific time points for efficacy assessment, e.g., 3 months, 6 months).
- Outcomes: Primary and secondary efficacy results (e.g., percentage reduction in striae width, Global Aesthetic Improvement Scale [GAIS] score improvement), methods of measuring improvement (e.g., Visual Analog Scale [VAS] for patient satisfaction, ultrasound for dermal thickness, histological analysis for collagen density), and reported side effects (type, incidence, and resolution time).

Quality Assessment of included studies was conducted using validated tools tailored to study design:

- RCTs: Assessed via the Cochrane Risk of Bias Tool (RoB 2.0), evaluating five critical domains: random sequence generation, allocation concealment, blinding of participants and personnel, blinding of outcome assessment, and handling of missing data. Only RCTs with “low risk” or “some concerns” in all critical domains were included.
- Observational studies (e.g., cohort studies, case–control studies): Evaluated using the Newcastle‐Ottawa Scale (NOS), which scores studies on three dimensions (selection of participants, comparability of groups, assessment of outcomes) with a maximum of 9 stars; studies with ≥ 7 stars were considered high quality.
- Meta‐analyses: Appraised using the AMSTAR 2 (A Measurement Tool to Assess Systematic Reviews) checklist, covering 16 items (e.g., protocol registration, comprehensive literature search, assessment of publication bias); meta‐analyses rated as “moderate” or “high” confidence were included.

## 3 Pathogenesis

The formation of SD is the result of the combined action of multiple pathophysiological processes. Its core mechanism involves the structural destruction and remodeling imbalance of the extracellular matrix (ECM) in the dermis. In recent years, studies have revealed that the formation of SD is the result of the involvement of multiple factors such as mechanical tension, hormonal abnormalities, genetic susceptibility, and inflammatory responses [1, 2, 13].

### 3.1 Mechanical Tension and Dermal Structure Damage

The mechanical stress caused by rapid skin expansion is one of the initiating factors for the occurrence of SD. The rapid expansion of the abdominal skin during pregnancy leads to the accumulation of mechanical stress, which exceeds the physiological tolerance range of the dermal tissue. This activates the mechanosensory pathway of fibroblasts, resulting in an imbalance in collagen/elastic fiber metabolism [14]. Histopathological examination shows that the elastin content in the SD area is lower than that in normal skin [7].

### 3.2 Abnormal Hormonal Regulation

Glucocorticoids reduce the skin's tensile resistance by receptor over‐expression, inhibiting fibroblast function and collagen metabolism. Both exogenous use and endogenous excess can significantly increase the risk [15]. Studies have shown that compared with normal skin, the estrogen receptors in the SD lesion area increase by 2.2 times, androgen receptors increase by 1.8 times, and glucocorticoid receptors (GR) increase by 1.7 times [16].

### 3.3 Genetic Susceptibility

A positive family history (mother/grandmother with SD) is a definite pathogenic factor. The proportion of positive family history in the case group is significantly higher than that in the control group [17, 18]. FN1 rs3910516 and ELN rs7787362 increase the susceptibility to SD by affecting the expression of fibronectin and the function of elastic fibers, respectively. SRPX (rs35318931), HMCN1 (rs10798036), and TMEM18 (rs7594220) increase the risk of SD by affecting the ECM or obesity‐related pathways. However, studies with different populations and sample sizes have shown inconsistent results [17, 18, 19].

### 3.4 Inflammatory Response

In the early stage of SD (Striae Rubrae stage), mast cell degranulation and macrophage activation are observed in the reticular layer of the dermis, accompanied by the rupture of elastic fibers and a decrease in ECM components (such as collagen, fibronectin FN, elastin ELN, fibrillin FBN) [20, 21]. The formation of stretch marks is directly related to the release of elastase by mast cells, which leads to the decomposition of elastic fibers. This process triggers a local inflammatory response, destroying the skin's elastic fiber network. The inflammatory response is accompanied by the infiltration of immune cells such as neutrophils and macrophages, further aggravating tissue damage [22, 23]. The immunoreactive activities of TLR2 and TLR4 are significantly enhanced in stretch marks. Through the MyD88‐dependent pathway (classical pathway), the levels of pro‐inflammatory cytokines such as TNF‐α are increased, promoting the formation of an inflammatory microenvironment and affecting the remodeling of the ECM [24]. The pathological process of stretch marks is similar to that of keloids and scleroderma, with abnormal expression of ECM‐related genes (such as collagen and elastin genes), which may be related to the dysregulation of inflammation‐related signaling pathways (such as TGF‐β) [24, 25].

## 4 Current Treatment Methods

The treatment methods for SD show a diversified development trend. According to the mechanism of action, they can be divided into four categories: topical drugs, physical therapy, energy devices, and combination therapies [2, 14]. Each method improves the imbalance of collagen metabolism and the disorder of the dermal matrix structure through different pathways, and there are significant differences in clinical efficacy and safety.

### 4.1 Topical Drug Treatment

As a basic treatment method, topical drugs mainly act by regulating ECM synthesis or inhibiting the inflammatory response.

Tretinoin promotes collagen synthesis and inhibits matrix metalloproteinases. After 12 weeks of single‐use, the collagen area increases significantly. It has a better effect on early red striae but a weaker effect on white striae. However, due to its limited skin permeability, the effective rate of monotherapy is relatively low, and it may cause irritating reactions such as erythema and desquamation [26, 27, 28].

Alpha hydroxy acids (AHAs) are widely used in SD management due to their exfoliative properties (loosening stratum corneum intercellular adhesions) and ability to stimulate fibroblast proliferation and collagen synthesis [2, 5]. Glycolic acid, a well‐studied AHA, can reduce the width of both new and older stretch marks by promoting collagen production, with noticeable improvements typically observed after 6 months of consistent use. Professional glycolic acid peels (higher concentrations) achieve deeper penetration and more significant results compared to at‐home formulations, making them a common clinical recommendation. Lactic acid, another common AHA, has been combined with topical tretinoin to enhance efficacy: combined use of tretinoin and lactic acid ointment can increase the content of elastic fibers in striae and improve the appearance of early striae, though this effect is less pronounced for mature striae. Additionally, Ud‐Din et al. [5] noted that AHAs (e.g., citric acid) are used in preventive care for SD during pregnancy, with regular application of 5% citric acid cream reducing the risk of Striae Rubrae development by improving skin barrier function—though large‐scale RCTs are still needed to confirm this preventive effect.

*Centella asiatica* extract improves stretch marks by stimulating fibroblast proliferation and collagen synthesis. It can promote the synthesis of type I collagen and enhance the repair of the extracellular matrix through the phosphorylation of the Smad2/Smad3 signaling pathway [29]. It shows good tolerance in combination therapies. In recent years, plant extracts such as green tea polyphenols [30] and tasmannia lanceolata leaf extract [31] have emerged due to their antioxidant and fibro‐remodeling promoting effects. After 8 weeks of treatment with a 3% green tea extract cream, the proportion of severe SD cases decreased from 86.1% (week 0) to 41.7%. Some studies have shown that regular use of olive oil in the third trimester of pregnancy can significantly reduce the risk of SD in primiparas and alleviate its severity, but the specific mechanism needs further verification [32].

However, topical drugs have limited improvement on mature white striae and often need to be combined with other treatment methods.

### 4.2 Physical Therapy Techniques

Microneedle technology has become an important option for SD treatment by triggering a wound–healing response through mechanical stimulation. Microneedle radiofrequency enhances dermal remodeling through heat energy superposition. Clinical data show that the newly formed collagen induced by it is better than that of NAFL [8, 33]. A meta‐analysis including 11 studies shows that the clinical improvement of the microneedle radiofrequency treatment group is more significant than that of the laser treatment group, but the postoperative pain is significantly higher than that of the laser group [9]. A latest study shows that a new microneedle combined with autologous platelet‐rich fibrin protocol has obvious improvements in the clinical effect, patient satisfaction, and tissue repair of stretch marks, suggesting that the combination of biological agents may break through the existing curative effect bottleneck [34].

Injectable Fillers are increasingly recognized as effective for atrophic Striae Albae, addressing the core pathological feature of dermal matrix loss by restoring volume and stimulating long‐term collagen synthesis [13]. As a biostimulatory filler, Calcium Hydroxylapatite (CaHA) acts as a scaffold to stimulate collagen and elastin production, plumping atrophic striae tissue and reducing visibility; it also promotes neovascularization, improving local blood flow and nutrient delivery to support tissue repair. Hyperdiluted CaHA (e.g., 1:1 dilution with normal saline) retains biostimulatory properties, facilitating collagen neogenesis and increasing dermal thickness, which is beneficial for improving striae appearance. Mechanistically, CaHA microspheres release calcium ions to activate calcium signaling and motor protein pathways (e.g., MYH9), enhancing fibroblast proliferation and collagen fiber orderly arrangement—showing superior regenerative effects compared to PMMA and PLLA fillers. Alsharif et al. [13] summarized clinical studies where intradermal CaHA injection improved striae depth and skin texture, with good biocompatibility and minimal inflammatory response. Cross‐linked Hyaluronic Acid (HA) fillers improve SD by restoring dermal hydration (critical for maintaining fibroblast function) and activating CD44‐mediated collagen synthesis. Seirafianpour et al. [14] included an RCT in their review where HA injection combined with microneedling enhanced therapeutic efficacy compared to HA monotherapy, likely due to microneedling creating microchannels that promote deeper HA penetration.

### 4.3 Energy Device Treatment

Energy devices achieve dermal structure remodeling by precisely regulating the depth of thermal damage and have become the core means of SD treatment.

#### 4.3.1 Ablative Fractional Laser

The CO_2_ fractional laser (10 600 nm) forms MTZs by vaporizing the epidermis to stimulate collagen regeneration. The CO_2_ fractional laser can significantly reduce the size (width/length) of the striae, improve skin texture, and has a high patient satisfaction. However, it has a poor effect on white striae. Its efficacy is similar to that of microneedle radiofrequency but with a higher cost, and the incidence of postoperative erythema and pigmentation is high [10, 35]. The Er: YAG laser (2940 nm) has fewer side effects (such as transient pigmentation) when treating stretch marks compared with other lasers [36].

#### 4.3.2 NAFL

Non‐ablative fractional laser (NAFL), represented by the 1565 nm erbium‐glass laser, has gained attention for its minimal invasiveness and ability to target the dermis without epidermal damage. This wavelength is selectively absorbed by water, penetrating to a depth of 1.5 mm to form microthermal zones (MTZs), which activate fibroblast proliferation and collagen remodeling [8, 11].

- Technical Principles and Efficacy: The 1565 nm NAFL's core innovation lies in its mid‐infrared wavelength (1550–1565 nm), which has a lower water absorption coefficient (5 cm^−1^) compared to ablative lasers like CO_₂_ (800 cm^−1^), allowing diffuse thermal effects rather than ablation [10]. This promotes collagen synthesis via heat shock protein (HSP70) and TGF‐β pathways while inhibiting MMP‐1 degradation [11]. Clinical evidence from randomized controlled trials (RCTs) robustly supports its efficacy and safety. Cao et al. [11] conducted a prospective RCT demonstrating that the combination of 1565 nm NAFL and topical β‐glucan led to a significant improvement in SD atrophy scores at the 12‐week follow‐up compared to monotherapy groups. Histological analysis corroborated these findings, showing increased epidermal thickness and enhanced collagen organization. In terms of safety, a study by Tang et al. [8] focusing on a Chinese population reported a low incidence of adverse events (4.8% experienced transient erythema), underscoring its high patient tolerance. These studies provide concrete scientific data supporting the clinical value of 1565 nm NAFL.
- Comparison with Other Modalities: While 1565 nm NAFL demonstrates advantages in safety and recovery time, it is important to note that microneedle radiofrequency (RF) may be slightly superior in terms of collagen hyperplasia and elastic fiber density, as highlighted by Tang et al. [8]. However, NAFL offers a better balance of efficacy and tolerance, making it suitable for patients requiring quick recovery, such as postpartum women. NAFL is effective for both striae rubrae and albae, with wide applicability across body areas [37].

This integrated summary avoids overemphasis on 1565 nm NAFL and provides a nuanced view within the broader context of energy‐based devices.

#### 4.3.3 Other Energy Devices

The pulsed dye laser (585 nm) targets the vascular abnormalities in early red striae and reduces the area of erythema through selective photothermal action [37]. The radiofrequency micro‐plasma technology generates a thermal effect by ionizing nitrogen and is superior to traditional microneedles in improving epidermal smoothness [38]. Non‐ablative 1540‐nm Er:Glass Laser is effective for Striae Rubrae, with clinical studies showing significant reductions in erythema and improvements in skin elasticity [12], 532 nm Nd:YAG Laser, a visible‐light laser that targets melanin and hemoglobin, making it effective for pigmented Striae Rubrae (Fitzpatrick skin types I‐III). It reduces vascularity and normalizes pigmentation, with clinical utility both as monotherapy and in combination with microneedling [37]. Picosecond Lasers, with ultra‐short pulse durations, induce non‐thermal mechanical damage to the dermis via the photoacoustic effect, stimulating collagen remodeling without excessive heat‐induced injury. This makes them suitable for dark skin types (Fitzpatrick IV‐VI) due to the low risk of pigmentation [14]. Intense Pulsed Light (IPL) uses broad‐spectrum light to target vascular and pigmented components of early Striae Rubrae. A meta‐analysis of clinical studies [37] demonstrates that IPL reduces erythema and improves skin texture, with a low adverse event rate.

### 4.4 Combination Therapies

#### 4.4.1 Laser Therapies Combined With PRP

Multiple studies [39, 40] showed that combining fractional CO_2_ laser with intradermal PRP injection resulted in significant clinical improvement (50%–75% improvement in most patients) compared to laser alone. Histological analysis revealed increased collagen and elastic fiber density. Patient satisfaction was higher with combination therapy, though transient side effects like erythema and hyperpigmentation were noted.

Research [41] demonstrated that Er:YAG laser combined with PRP improved striae albae better than laser alone, with enhanced epidermal thickness and reduced atrophy. Pain was moderate, but no severe adverse effects were reported.

#### 4.4.2 Microneedling Combined With PRP or Other Agents

Several studies [42, 43] found that microneedling followed by PRP application led to marked improvement in striae rubrae and striae albae, with reduced width and improved texture. Patient satisfaction scores were high (86.6% very satisfied). Dermoscopy showed characteristic changes including reduced hypopigmented areas and enhanced perivascular signals, which are indirect markers of improved dermal microenvironment and tissue repair; these dermoscopic features were consistent with histological evidence of increased collagen deposition and improved vascularity in the same studies [42, 43].

Ebrahim et al. [44] compared microneedling with topical hyaluronic acid versus insulin; both showed improvement, but insulin combined with microneedling had slightly better results in epidermal density enhancement.

#### 4.4.3 Radiofrequency and Laser Combinations

Research [45, 46] indicated that combining fractional microneedle radiofrequency (FMR) with fractional CO_2_ laser provided superior clinical improvement in SD compared to either treatment alone. Ultrasound and optical coherence tomography (OCT) showed increased dermal thickness and collagen density. However, FMR had a lower risk of post‐inflammatory hyperpigmentation (PIH) than CO_2_ laser.

#### 4.4.4 Other Combination Therapies

Several studies [10, 47] compared carboxytherapy with fractional CO_2_ laser; both improved SD, but laser showed better results in reducing striae width and improving skin texture. Ebrahim et al. [48] found that combining PRP with subcision or medium‐depth peels (e.g., glycolic acid 70% + trichloroacetic acid 35%) resulted in higher patient satisfaction and greater histological improvement in collagen organization than PRP alone. A meta‐analysis [33] compared fractional microneedle radiofrequency (FMR) and fractional CO_2_ laser for SD. Both were effective, but FMR had a significantly lower risk of PIH (OR: 0.24). Clinical improvement and patient satisfaction were similar between groups. Another systematic review [14] concluded that combination therapies (e.g., laser + PRP) generally outperformed monotherapies in terms of efficacy and patient‐reported outcomes.

## 5 Discussion

Although significant progress has been made in the field of SD treatment, many challenges still remain. Firstly, the problems of heterogeneous efficacy and individual differences are prominent. Most existing studies are based on small samples [11, 12, 49], and the heterogeneity of different device parameters and treatment plans (single‐use or combination) leads to poor comparability of results [9, 50]. For example, microneedle radiofrequency and CO_2_ fractional laser have similar effects on collagen remodeling for improving white striae, but microneedle radiofrequency causes more significant postoperative pain [8, 51]. Moreover, the risk of pigmentation is higher in patients with dark skin after laser treatment [10, 33]. In addition, the effects of factors such as patient age, skin type, and SD stage (red striae/white striae) on the curative effect have not been fully clarified [36], and there is a lack of stratified treatment strategies for different subgroups.

Secondly, the lack of standardization of the evaluation system restricts the reliability of research. Currently, the evaluation of curative effects mostly depends on subjective scores by physicians (such as GAIS) or self‐evaluation by patients (VAS) [2, 52], and only a few studies use ultrasonic measurement of dermal thickness or histological analysis [53, 54]. In addition, there is a lack of long‐term follow‐up data [12]. Most studies only observe for 3–6 months [52, 55], making it impossible to evaluate the recurrence risk.

At the technical level, the synergistic mechanism of combination therapies needs to be clarified. Although the combination of 1565 nm NAFL with β—β‐glucan or PRP can enhance collagen regeneration [11, 40], the optimal combination mode and target sites have not been determined [14, 54]. Although cold atmospheric plasma technology [56] and plant extracts [31] have shown initial efficacy, their molecular mechanisms (such as the regulation of the TGF—β signaling pathway) still need to be further explored.

Future research should focus on the following directions:

- Large‐sample long‐term studies: Verify the durability and safety of existing therapies through multi‐center randomized controlled trials [12], especially paying attention to the treatment responses of dark‐skinned populations and special areas [55, 57].
- Standardized evaluation system: Integrate subjective scores, biomechanical parameters, and imaging markers to establish a multi‐dimensional efficacy evaluation standard.
- Technological innovation and combination strategies: Optimize NAFL parameters to balance efficacy and safety; develop precision treatments based on stem cells [54].
- Artificial intelligence‐assisted diagnosis and treatment: Analyze clinical data through machine learning to predict individualized treatment plans and establish risk–stratification models to guide early intervention.

In summary, the treatment of SD needs to break through the existing technical bottlenecks, integrate basic research and clinical transformation, and ultimately achieve the transformation from “improving appearance” to “biological repair”.

## 6 Conclusion

The treatment of SD has shifted from traditional local medication to an individualized combination strategy dominated by energy devices. Fractional microneedling plus PRP and fractional CO₂ laser resurfacing with PRP are both used with good results and good patient satisfaction in SD rubra and alba [39, 40, 42, 43], 1565 nm NAFL promotes dermal collagen remodeling through precise thermal stimulation and shows significant advantages in improving atrophic striae, epidermal thickness, and pigment uniformity [8, 11, 58]. Combination therapies (such as NAFL combined with β‐glucan or PRP) can further enhance the synergistic efficacy [11, 29, 40]. In the future, it is necessary to optimize the precise matching of energy‐depth‐interval through multi‐center studies and explore artificial‐intelligence‐based personalized plans.

## Author Contributions

Yi Wu: literature review, writing, and original draft preparation. Huaigu Wang: supervision, review and editing.

## Conflicts of Interest

The authors declare no conflicts of interest.

## Data Availability

The data that support the findings of this study are available on request from the corresponding author. The data are not publicly available due to privacy or ethical restrictions.
